# Optimization of the image contrast for the developing fetal brain using 3D radial VIBE sequence in 3 T magnetic resonance imaging

**DOI:** 10.1186/s12880-022-00737-1

**Published:** 2022-01-20

**Authors:** Yi Liao, Xuesheng Li, Fenglin Jia, Zhijun Ye, Gang Ning, Sai Liu, Pei Li, Chuan Fu, Qing Li, Shaoyu Wang, Huapeng Zhang, Haibo Qu

**Affiliations:** 1 Department of Radiology, West China Second University Hospital of Sichuan University, Chengdu, Sichuan People’s Republic of China; 2 Key Laboratory of Birth Defects and Related Diseases of Women and Children (Sichuan University), Ministry of Education, West China Second University Hospital, Sichuan University, Chengdu, Sichuan People’s Republic of China; 3 MR Collaborations, Siemens Healthineers, Shanghai, People’s Republic of China; 4 MR Scientific Marketing, Siemens Healthineers, Shanghai, People’s Republic of China; 5 MR Application, Xi’an Branch of Siemens Healthineers, Shanxi, People’s Republic of China

**Keywords:** Fetal brain, Radial VIBE sequence, MRI, Image quality, Development

## Abstract

**Background:**

Faster and motion robust magnetic resonance imaging (MRI) sequences are desirable in fetal brain MRI. T1-weighted images are essential for evaluating fetal brain development. We optimized the radial volumetric interpolated breath-hold examination (VIBE) sequence for qualitative T1-weighted images of the fetal brain with improved image contrast and reduced motion sensitivity.

**Materials and methods:**

This was an institutional review board-approved prospective study. Thirty-five pregnant subjects underwent fetal brain scan at 3 Tesla MRI. T1-weighted images were acquired using a 3D radial VIBE sequence with flip angles of 6º, 9º, 12º, and 15º. T1-weighted images of Cartesian VIBE sequence were acquired in three of the subjects. Qualitative assessments including image quality and motion artifact severity were evaluated. The image contrast ratio between gray and white matter were measured. Interobserver reliability and intraobserver repeatability were assessed using intraclass correlation coefficient (ICC).

**Results:**

Interobserver reliability and intraobserver repeatability universally revealed almost perfect agreement (ICC > 0.800). Significant differences in image quality were detected in basal ganglia (*P* = 0.023), central sulcus (*P* = 0.028), myelination (*P* = 0.007) and gray matter (*P* = 0.023) among radial VIBE with flip angles 6º, 9º, 12º, 15º. Image quality at the 9º flip angle in radial VIBE was generally better than flip angle of 15º. Radial VIBE sequence with 9º flip angle of gray matter was significantly different by gestational age (GA) before and after 28 weeks (*P* = 0.036). Quantified image contrast was significantly different among different flip angles, consistent with qualitative analysis of image quality.

**Conclusions:**

Three-dimensional radial VIBE with 9º flip angle provides optimal, stable T1-weighted images of the fetal brain. Fetal brain structure and development can be evaluated using high-quality images obtained using this angle. However, different scanners will achieve different TRs and so the FA should be re-optimized each time a new protocol is employed.

## Introduction

As a non-invasive medical imaging technique with high resolution and non-ioninzing radiation, magnetic resonance imaging (MRI) has been widely used in assessment of the fetus [1]. Magnetic resonance imaging (MRI) of the fetal brain provides a safe and powerful way to examine the brain anatomy and diseases during early development. Fetal MRI has been widely applied to investigate structural abnormalities of the fetal brain, such as ventricular dilatation, cerebellar dysplasia, and neuronal migration disorder [2]. The sequences generally used for fetal brain MRI are fast T2-weighted imaging(T2WI), T1-weighted imaging(T1WI), and diffusion weighted imaging (DWI). However, fetal brain images are known to have poor contrasts that are challenging for anatomical definition and detection of abnormalities. The main factors underlying this difficulty are inadequate contrast and artifacts associated with respiration of pregnant mother and movement of the fetus. It has been considered difficult to produce high-quality T1WI images of the fetal brain. T1WI contrast may be used to visualize regions of protein, calcification, hemorrhage, and fat. Furthermore, it is also capable of displaying brain structures and the changes from hypo- to hyper-intensity to reflect the maturation of myelin to some degree. Myelination, lamination, and migration are essential in brain development. These processes must occur correctly to form normal brain structure. The formation of normal brain structures, such as basal ganglia, central sulcus, lateral fissure, optic chiasm, gray matter, as well as myelination, reflect normal brain developmental processes, including lamination, migration, and myelination. Myelination is associated with decreased T1 relaxation time, which changes from hypointense to hyperintense relative to gray matter on T1WI. Thus, the T1WI sequence can be used to assess whether brain maturation is progressing normally [3]. Specific milestones have been established for when changes in white matter intensity (relative to gray matter intensity) are expected. Therefore, finding high-resolution T1WI by which to assess brain structure and level of myelination is important for assessment of the fetal nervous system. However, due to unpredictable fetal movement and abdominal motion of the pregnant mother, the scanning sequence and parameters are complicated and need to be appropriately defined.

Efforts have been made to maximize T1-weighted image contrast in the fetal brains. Gholipour et al. conducted a thorough review of fetal MRI imaging techniques. Various acquisition schemes are used for fetal T1-weighted imaging [4]. Inversion recovery (IR) pulse-prepared single-shot turbo-spin-echo (TSE) acquisition exhibits high image quality and robustness to fetal movements and maternal breathing motion. Although a long echo train could be used to shorten acquisition time, this would increase T2 blurring. The specific absorption ratio (SAR) is another concern in the TSE sequence, especially at 3 T scanner. Gradient-echo based acquisition can significantly reduce SAR. However, this approach may be limited from either mixed weighting of T1 and T2* for spoiled acquisition or banding artifact for balanced acquisition [4]. To enhance T1 contrast, an IR preparation pulse is applied to gradient sequences [5]. To decrease motion artifacts while imaging the fetus and eliminate the need for sedation, T1-weighted images are typically acquired through a two-dimensional turbo fast low-angle shot (FLASH) sequence. FLASH uses radio frequency excitation pulses with a low flip angle (less than 90º) and subsequent reading gradient reversal to produce a gradient echo signal [6]. In multi-shot gradient-echo sequences all slices are acquired sequentially. Subsequently, given slight fetal movement at any time during the acquisition period, all slices are degraded by motion artifacts. Therefore, single-shot acquisition is widely applied to minimize motion artifact in 2D scans. Inversion-recovery-prepared half-Fourier acquisition single-shot turbo spin echo (IR-HASTE) acquisition is another choice for T1 imaging. IR-HASTE is based on single-slice acquisition. Fetal motion typically affects the particular slice that is being acquired while motion occurs [7]. The use of large echo train lengths with short echo time (TE) results in blurring and loss of contrast. Scan efficiency is improved to directly increase robustness against motion in 2D fetal MRI. 3D images are reconstructed using three-plane post reconstruction. An alternative way to reduce motion sensitivity is to use motion insensitive encoding trajectories, such as radial acquisition. One of the clinically available sequences is radial non-Cartesian T1-weighted gradient-recalled echo, known as radial VIBE (volumetric interpolated breath-hold examination, named radial VIBE by Siemens Healthcare as product sequence) [8–10]. This sequence provides several advantages over conventional 3D T1-weighted GRE imaging in its robustness to pregnant women’s respiratory motion and unpredictable motion by the fetus [11]. The radial acquisition minimizes breathing motion. Due to the radial acquisition pattern, some of the motion artifacts are spread out in the image space, which would be less noticeable.

Therefore, to reduce motion artifacts, a 3D radial VIBE sequence [12] was used and optimized in our study. Because the T1 values in white matter and gray matter differ between fetuses and newborns, and T1 values change with development of the fetal brain, imaging parameters could be optimized to provide better T1 contrast for the fetal brain. The T1 contrast of the 3D radial VIBE sequence primarily depends on imaging parameters, including flip angle (FA) and repetition time (TR). In our research, the shortest TR was utilized to minimize scan time. Therefore, different flip angles were utilized to find the most suitable parameter for the 3D radial VIBE sequence and compared with the Cartesian VIBE sequence.

## Materials and methods

### Participants

Thirty-five pregnant subjects were recruited through poster advertisements for fetal brain MRI scan in West China Second University Hospital of Sichuan University. Thirty-two of these subjects were scanned for radial VIBE sequence and three subjects for both radial VIBE sequence and cartesian VIBE sequence. They were healthy middle-term or late-term pregnant women with gestational age (GA) from 25 to 38 weeks. The average gestational age was 30 weeks and 4 days. No participants had a history of significant systemic or neurological illness. In addition, there was no known history of major psychiatric illness in their first-degree relatives. All participants were from a similar geographical region. The following exclusion criteria applied to participate: (1) history of substance abuse, or (2) major physical illness, such as cardiovascular disease or hepatitis, as assessed by clinical evaluations and medical records. The institutional ethics committee approved the study. All participants provided written informed consent to participate.

### Data acquisition

MRI examinations were performed on a 3 T MR scanner (MAGNETOM Skyra, Siemens Healthcare) with an 18-channel phased-array coil. T1-weighted images were acquired using a 3D radial stack-of-star GRE sequence (StarVIBE, a Siemens product sequence) for free breathing. Radial VIBE sequence was performed 4 times with different flip angles: 6º, 9º, 12º, and 15º, the acquisition parameters were as follows: slice thickness = 2.0 mm, TR = 3.86 ms, TE = 1.65 ms, voxel size = 1.6 × 1.6 × 2.0 mm^3^, field of view = 350 × 350 mm^2^. The acquisition time was 1 min and 7 s for each measurement. As a comparison, Cartesian VIBE images were acquired for free breathing with the following scanning parameters: slice thickness = 2.0 mm, TR = 3.86 ms, TE = 2.46 ms, voxel size = 1.6 × 1.6 × 2.0 mm^3^, field of view = 350 × 350 mm^2^. The acquisition time was 27 s for each measurement.

### Image analysis

The acquired 3D radial VIBE images were divided into four groups with different imaging parameters: flip angles of 6º, 9º, 12º, and 15º. Image quality and motion artifacts were assessed and scored by three experienced radiologists. The image contrast ratio between gray and white matter were calculated as a metric to quantify the image contrast. Thus, the performance of different protocols was evaluated and compared via qualitative analysis and quantitative analysis.

### Qualitative image analysis

Qualitative image quality analysis was performed independently by three radiologists (Yi Liao, Fenglin Jia, Haibo Qu; 11 years, 11 years, and 16 years of experience in MRI diagnosis, respectively). The graders were not informed of the acquisition parameters.

Image quality was evaluated as the contrast and visualization of basal ganglia, central sulcus, lateral fissure, optic chiasm, and gray matter, as well as myelination. Image quality of normal anatomic structures was evaluated by means of a five-point scale. Motion artifacts during the data acquisition degraded the image quality. Thus, artifacts and noise were evaluated and assessed in each group using a four-point scale. The graders considered the graininess and blurriness in the images as noise. Three radiologists independently scored the image quality and artifacts based on the criteria listed in Table 1. Image quality assessment was conducted in three pregnant subjects in both radial VIBE and Cartesian VIBE by expression.

**Table 1:** The scores for image quality and artifacts

Score	Image quality	Artifacts
0	Unable to perceive the anatomy of the boundary of gray matter and white matter, myelination, and sulcus, rendering the image nondiagnostic	Severe artifact present (underlying anatomy could not be visualized)
1	Inadequate display of anatomy of the boundary of gray matter and white matter, myelination, and sulcus	Moderate artifact present (underlying anatomy could be visualized but delineation was suboptimal)
2	Subpar image quality of the boundary of gray matter and white matter, and myelinization, and the sulcus vaguely displayed, rendering the image inadequate for diagnosis	Mild artifact present (underlying anatomy well-visualized)
3	Visible boundary of gray matter and white matter, and myelinization, with the sulcus vaguely displayed, insufficient soft tissue contrast, but overall acceptability for diagnosis	No artifact
4	Visible boundary of gray matter and white matter, and myelinization, well-defined sulcus, and high soft tissue contrast, rendering the image adequate for diagnosis	–

### Quantitative image analysis

For quantitative image analysis, one radiologist performed intensity measurements. The intensity of each subject's T1-weighted image was scaled to a range of 0 to 300. Regions of interest (ROIs) were placed in bilateral basal ganglia and bilateral deep white matter of the frontal lobe, both with the same area of 10.15 mm^2^. Then, the intensity within the ROIs was measured at the same window level of 267 and window width of 500 by RadiAnt DICOM Viewer (version 2020.1, Medixant, Poland). Image contrasts between gray and white matter were calculated from the intensity measurement in different protocols (flip angles of 6 º, 9 º, 12 º, and 15º in radial VIBE sequences).

### Statistical analysis

Statistical analysis was performed using SPSS (version 22, IBM Corporation, New York, USA) and GraphPad Prism software (version 7, GraphPad Software, Inc., USA). Image quality scores in brain regions (basal ganglia, central sulcus, lateral fissure, optic chiasm, gray matter, myelination) were compared among different protocols (3D radial VIBE with flip angles of 6º, 9º, 12º, and 15º) using a Kruskal–Wallis test. Then, post-hoc two-sample tests were conducted using Dunn’s multiple comparison. To test for image quality differences in brain regions between GA before 28 weeks and after 28 weeks, a Mann–Whitney U test was performed. Image contrast among protocols was compared using ANOVA, after testing for a Gaussian distribution. For all tests, *P* < 0.05 indicated a significant difference.

In addition, interobserver reliability and intraobserver repeatability of assessments of image quality among protocols was calculated using the intraclass correlation coefficient (ICC) according to McGraw and Wong [13], applying a two-way mixed-model and one-way model, respectively. The main observer reanalyzed image quality after 1 month in randomized order, to evaluate intraobserver repeatability. ICC was interpreted as follows: a value less than 0.20 indicated poor agreement, 0.21–0.40 indicated fair agreement, 0.41–0.60 indicated moderate agreement, 0.61–0.80 indicated substantial agreement, and 0.81–1.00 indicated almost perfect agreement [14].

## Results

**Fig. 1:**
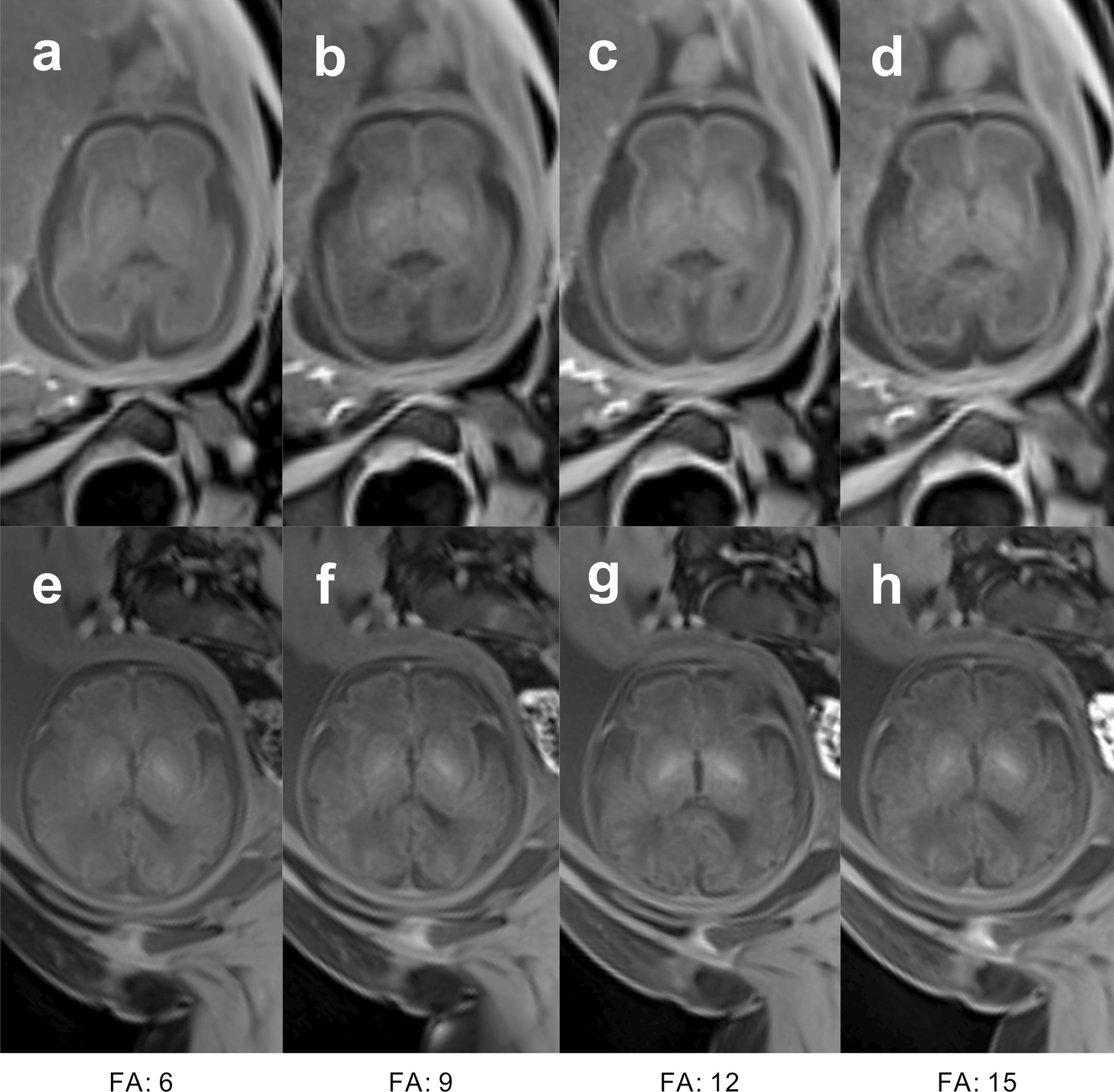
Fetal brain images of 27-week (**a**–**d**) and 32-week (**e**–**h**) pregnant women, obtained using 3D radial VIBE sequences with flip angles of 6º (**a**, **e**), 9º (**b**, **f**), 12º (**c**, **g**), and 15º (**d**, **h**)

A total of 32 cases were available for analysis. Radial VIBE with a 9º flip angle of two subjects, radial VIBE with a 15º flip angle of one subject were scanned twice. Other subjects with different protocols were scanned once. Figure 1 shows normal fetal brain images obtained from 27-week and 32-week pregnant women using 3D radial VIBE sequences with flip angles of 6º, 9º, 12º, and 15º.

### The interobserver reliability analysis and the intraobserver repeatability analysis

The interobserver reliability analysis demonstrated substantial agreement in evaluation of basal ganglia and myelination using radial VIBE with 15º flip angle (ICC = 0.7795, ICC = 0.7505 respectively). Other conditions were associated with almost perfect agreement (ICC > 0.8000). The intraobserver repeatability analysis showed almost perfect agreement (ICC > 0.8000) in all brain regions with different protocols.

### Image quality analysis

Table 2 summarizes differences in image quality, as evaluated by Kruskal–Wallis test. There were significant differences among protocols (3D radial VIBE sequence with flip angles of 6º, 9º, 12º, and 15º) in each brain region. The pairwise post-hoc comparison of image quality by Dunn’s multiple comparison is shown in Table 3. Additionally, the 3D radial VIBE sequence with 9º flip angle exhibited better image quality than did the15º flip angle in basal ganglia, central sulcus, myelination, and gray matter.

**Table 2:** Image quality of different protocols (3D RadialVIBE sequence with flip angles of 6º, 9º, 12º, and 15º) compared by Kruskal–Wallis test

Brain region	*P* value	Kruskal–Wallis statistic value
Gray matter	0.025	9.367
Basal ganglia	0.022	9.647
Central sulcus	0.042	8.187
Myelination	0.013	10.79
Optic chiasm	0.138	5.519
Lateral fissure	0.022	9.647

**Table 3:** Post-hoc two-sample test of image quality differences in different fetal brain regions, by Dunn’s multiple comparison

Sequence	Sequence	Basal ganglia		Central sulcus		Myelination		Optic chiasm		Lateral fissure		Gray matter	
		Mean difference	*P* value	Mean difference	*P* value	Mean difference	*P* value	Mean difference	*P* value	Mean difference	*P* value	Mean difference	*P* value
FA9	FA15	26.03	0.023*	24.95	0.028*	28.94	0.007*	18.64	0.130	22.98	0.063	26.03	0.023*

*significant difference

The image quality of 3D radial VIBE with a 9º flip angle did not differ significantly by Mann–Whitney U test between GA before 28 weeks and GA after 28 weeks in each region of the basal ganglia (*P* = 0.476), myelination (*P* > 0.999), central sulcus (*P* = 0.583), lateral fissure (*P* = 0.514), and optic chiasm (*P* = 0.901). The image quality of gray matter (*P* = 0.036) was significantly different between GA before 28 weeks and GA after 28 weeks.

### Noise and artifact analysis

Kruskal–Wallis tests revealed no differences in noise and artifact among the 3D radial VIBE sequence with flip angles of 6º, 9º, 12º, and 15º (*P* > 0.05).

### Quantitative assessments of image contrast

**Fig. 2:**
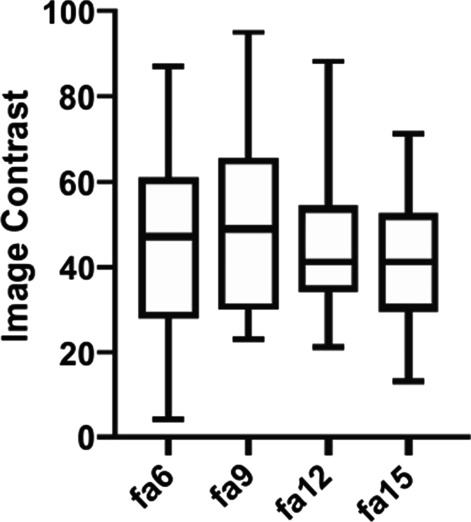
Image contrast of 3D radial VIBE sequences with flip angles of 6º, 9º, 12º, 15º. The image contrast is the ratio between gray and white matter

Analysis of variance (ANOVA) revealed quantitative assessments of image contrast differed significantly among 3D radial VIBE with flip angles of 6º, 9º, 12º, and 15º (F = 3.916, *P* = 0.011). Figure 2 shows the results of Fishers’ least significant difference test, which was conducted as a post-hoc test following ANOVA. In addition, image contrast was better with a 9º flip angle than with a 15º flip angle.

### Qualitative analysis of radial VIBE and Cartesian VIBE

**Fig. 3:**
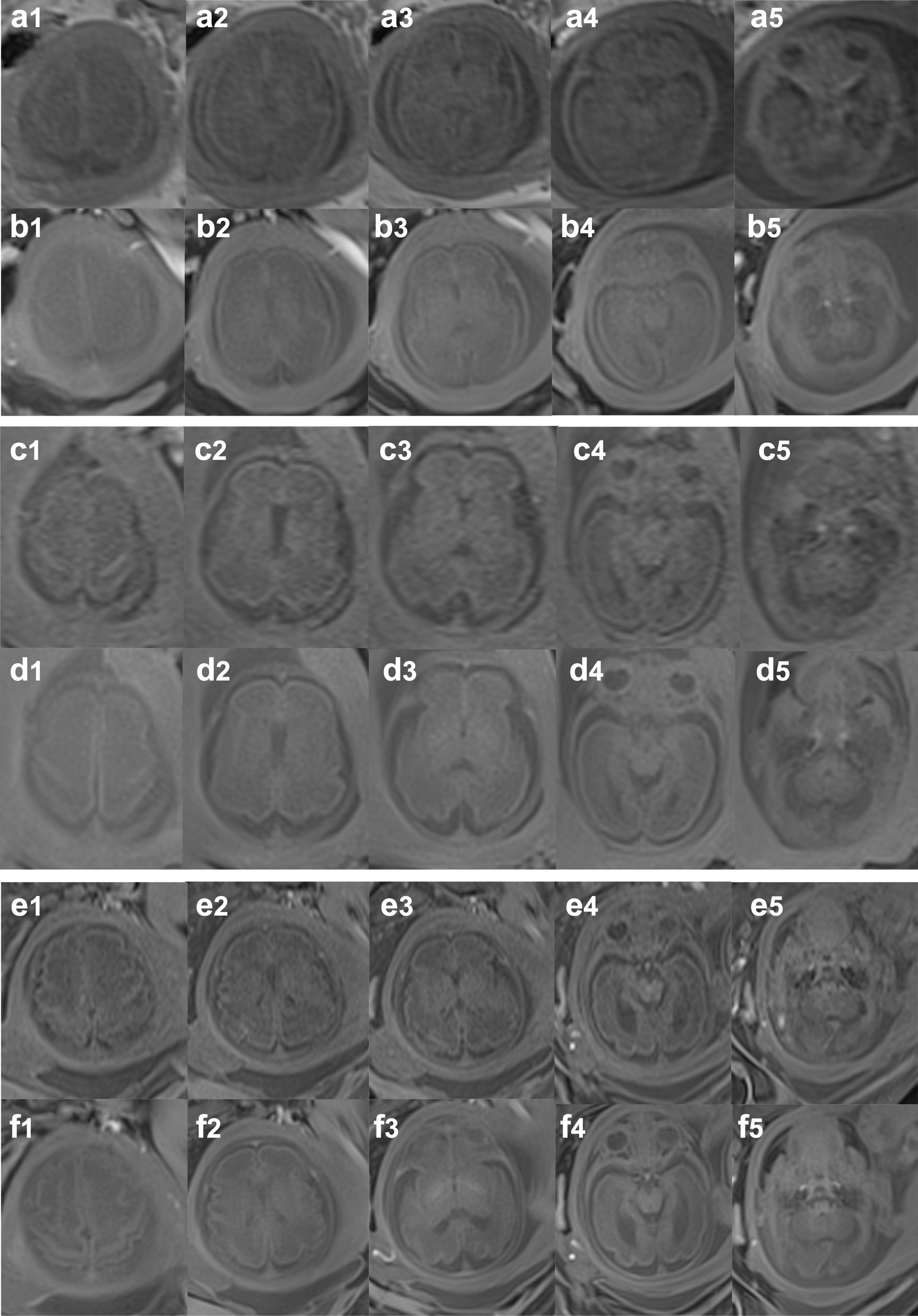
Fetal brain images of 26-week (**a**, **b**), 28-week (**c**, **d**), and 34-week (**e**, **f**) pregnant women, obtained using Cartesian VIBE sequence (a, c, e) and 3D radial VIBE sequence (**b**, **d**, **f**) in free breathing

**Fig. 4:**
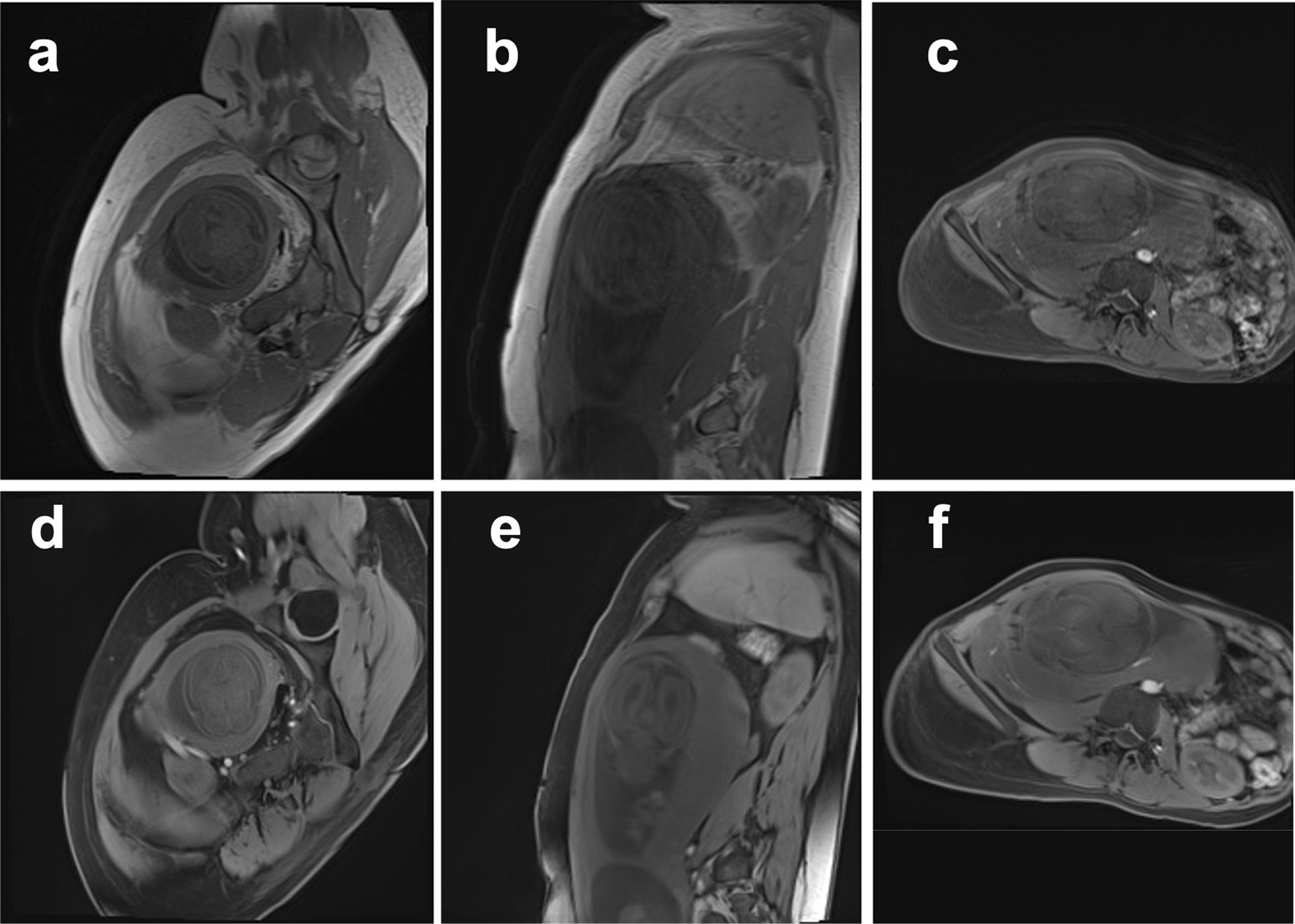
Full field of view images of 26-week (**a**, **d**), 28-week (**b**, **e**), and 34-week (**c**, **f**) pregnant women obtained using Cartesian VIBE sequence (**a**–**c**) and 3D radial VIBE sequence (**d**–**f**) in free breathing

The status of radial VIBE and Cartesian VIBE sequences were the same except for the different acquisition trajectories. They were both collected in the free-breathing state. From the data collected with matched resolution and contrast properties from three subjects, the images of radial VIBE sequence had better anatomical image contrast, showed clearer anatomical structures, less noise, and fewer motion artifacts compared to the images of Cartesian VIBE sequence. Figure 3 showed the images of fetal brain acquired by radial VIBE and Cartesian VIBE of three subjects. Figure 4 showed full field of view images of three subjects to appreciate breathing artifacts comparing cartesian VIBE vs radial VIBE.

## Discussion

In this study, a 3D radial VIBE sequence with different flip angles was utilized to image the fetal brain. Three of these images were compared with those obtained using Cartesian VIBE sequence. A 3D radial VIBE sequence with a flip angle of 9º provided the best T1 contrast for displaying fetal brain structures over gestational ages from 25 to 38 weeks. Image quality of brain structures obtained using 3D radial VIBE with a 9º flip angle did not differ significantly between GA before 28 weeks and GA after 28 weeks.

Motions from fetal movements and respiratory of pregnant women usually blurs the images in traditional Cartesian acquisitions. Respiratory motion remains a major challenge in MRI, particularly for abdominal and cardiovascular imaging. Due to the limited encoding speed in conventional MRI, k-space lines may be acquired in different respiratory motion states during free-breathing, resulting in ghosting artifacts and image blurring. The simplest approach to avoid respiratory motion effects is to suspend respiration during data acquisition. However, it’s hard to perform breath-hold acquisitions in pregnant women. Non-Cartesian k-space sampling schemes, such as radial or spiral, are less sensitive to respiratory motion and enable free-breathing imaging at the expense of increased scan times [15]. The nice property of these sequences is in fact that they can be retrospectively corrected for semi-periodic motion (i.e., maternal breathing). Point Spread Function (PSF) properties of radial VIBE are much better than Cartesian VIBE, and that while in the former motion would simply blur the data if the field of view was chosen to be sufficiently large, in the latter motion creates coherent artifacts potentially covering the fetal head. The 3D radial VIBE sequence is a radial non-Cartesian T1-weighted gradient-recalled echo sequence that helps reduce motion artifacts. Three-dimensional radial VIBE sequence is with non-Cartesian radial acquisition which increases its robustness to motion [12, 16]. For radial k-space trajectories, motion artifacts are effectively spread throughout the entire field of view (FOV) [17]. Artifacts from motion are less noticeable in radial acquisition than in the Cartesian methods. Since the geometry of radial scanning enables oversampling along readout direction, no aliasing effect will occur if the FOV is properly selected.

Radial sampling has proven to be very useful and especially suitable for populations such as pregnant women [18, 19]. Respiratory motion is more disturbing to imaging if the fetus is in breech presentation. Even if the fetus remains still, head motion may occur in breech presentation, in which the head lies close to maternal diaphragm; maternal respiratory motion is directly transmitted to the head of the fetus. Non-Cartesian radial acquisition enables imaging the fetus without requiring the pregnant woman to hold her breath. Breathing artifacts in our pregnant participants had little impact on the image quality of the fetal brain; high-quality images of the fetal brain were obtained without breath holding or sedation. This acquisition scheme makes radial VIBE optimal for fetal brain scans.

In this study, the imaging parameters of the radial VIBE sequence was optimized for the improved T1-weighted contrast of the developing fetal brain. The image at radial VIBE with flip angle 9º outperforms images acquired with other protocols in both middle-term and late-term pregnancy. T1 relaxation time shortens as the development of fetal brain. To estimate the stability of the performance of radial VIBE with flip angle 9 º, the image qualities were compared between GA > 28 weeks and GA < 28 weeks. Mann–Whitney U test suggests there is no significant difference between the images at middle-term and late-term pregnancy. Moreover, as myelination developed with GA, artifact and noise at different GAs remained stable in the radial VIBE sequence with 9º flip angle. The 3D VIBE sequence has been utilized to assess the normal and abnormal fetal gastrointestinal tract [20]. Radial VIBE sequence has been reported with motion robustness for swallowing and eye movements. Therefore, radial VIBE sequence with a 9º flip angle and short TR could be beneficial in imaging fetal brain.

In this study, fetal brain structures and myelination were clearly shown by the 3D radial VIBE sequence. These structures reflect the development of the fetal brain, including lamination, migration, and myelination. Additionally, these images provide a foundation for revealing pathological signals. During the fetal period, calcification may be detected in congenital intracranial infections and hemorrhage may occur for various reasons. Hemorrhage and calcification are reflected in hyperintense regions via T1WI sequence. Ventricular cysts and arachnoid cysts are reflected as hypointense regions via this sequence. Pathological manifestations and abnormal signal changes could be located precisely given clear imaging of normal brain structure. The assessment of normal brain development and detection of abnormal signals are important for ensuring optimal pregnancy outcome.

Our study has some limitations. We enrolled a limited number of fetuses with normal brain development on MRI scan. There was no segmentation of the structures which was subjective to the assessment. Also, the myelination assessment was subjective by detecting the T1WI hypersignals. In the future, the quantitative assessment of myelination is the direction of ongoing research. The spatial distribution of B1 + is inhomogeneous. However, our study has focused on studying the impact of the norminal flip angle and ignored the impact of spatial variations of the B1 field. What’s more, the impact of VIBE on findings and diagnosis in cases of abnormal brain development remains unclear. Therefore, further studies of abnormal fetal brains should be conducted, using 3D radial VIBE sequences. Future studies with larger samples are needed to evaluate the potential role of the radial VIBE sequence in the detection of fetal brain pathology.

## Conclusion

In conclusion, image quality and artifact assessment of normal fetal brain structure and development was conducted using 3D radial VIBE T1WI sequences with flip angles of 6º, 9º, 12º, 15º, and Cartesian VIBE T1 sequences in free breathing. A 3D radial VIBE sequence with a 9º flip generated optimal T1WI images of the fetal brain. 3D radial VIBE exhibited few artifacts associated with maternal and fetal movements. The performance of the 3D radial VIBE contrast is stable during pregnancy. In this study, fetal brain structure and development can be evaluated using high-quality images obtained using a 3D radial VIBE sequence with a 9º flip angle. However, different scanners will achieve different TRs and so the FA should be re-optimized each time a new protocol is employed.

## Data Availability

The datasets used and/or analyzed during the current study are available from the corresponding author on reasonable request.

## Abbreviations

VIBE: Volumetric interpolated breath-hold examination
FLASH: Fast low-angle shot
ICC: Intraclass correlation coefficient
GA: Gestational age
MRI: Magnetic resonance imaging
T2WI: T2-weighted imaging
T1WI: T1-weighted imaging
DWI: Diffusion weighted imaging
IR: Inversion recovery
TSE: Turbo-spin-echo
SAR: Specific absorption ratio
IR-HASTE: Inversion-recovery-prepared half-Fourier acquisition single-shot turbo spin echo
TE: Echo time
FA: Flip angle
TR: Repetition time
FOV: Field of view
TI: Inversion time
ANOVA: Analysis of variance

## References

[1] Saleem SN (2014). Fetal MRI: an approach to practice: a review. J Adv Res.

[2] Glenn OA, Barkovich AJ (2006). Magnetic resonance imaging of the fetal brain and spine: an increasingly important tool in prenatal diagnosis, part 1. Am J Neuroradiol..

[3] Barkovich AJ (2000). Concepts of myelin and myelination in neuroradiology. Am J Neuroradiol.

[4] Gholipour A, Estroff JA, Barnewolt CE, Robertson RL, Grant PE, Gagoski B, Warfield SK, Afacan O, Connolly SA, Neil JJ (2014). Fetal MRI: a technical update with educational aspirations. Concepts Magn Reson Part A.

[5] Ferrazzi G, Price AN, Teixeira RP, Cordero-Grande L, Hutter J, Gomes A, Padormo F, Hughes E, Schneider T, Rutherford M (2018). An efficient sequence for fetal brain imaging at 3T with enhanced T1 contrast and motion robustness. Magn Reson Med.

[6] Huber AM, Schoenberg SO, Hayes C, Spannagl B, Engelmann MG, Franz WM, Reiser MF (2005). Phase-sensitive inversion-recovery MR imaging in the detection of myocardial infarction. Radiology.

[7] Tang Y, Yamashita Y, Namimoto T, Takahashi M (1998). Characterization of focal liver lesions with half-Fourier acquisition single-shot turbo-spin-echo (HASTE) and inversion recovery (IR)-HASTE sequences. J Magn Reson Imaging.

[8] Chandarana H, Block KT, Winfeld MJ, Lala SV, Mazori D, Giuffrida E, Babb JS, Milla SS (2014). Free-breathing contrast-enhanced T1-weighted gradient-echo imaging with radial k-space sampling for paediatric abdominopelvic MRI. Eur Radiol.

[9] Shin HJ, Kim MJ, Lee MJ, Lala SV, Mazori D, Giuffrida E, Babb JS, Milla SS (2016). Comparison of image quality between conventional VIBE and radial VIBE in free-breathing paediatric abdominal MRI. Clin Radiol.

[10] Fujinaga Y, Ohya A, Tokoro H, Yamada A, Ueda K, Ueda H, Kitou Y, Adachi Y, Shiobara A, Tamaru N (2014). Radial volumetric imaging breath-hold examination (VIBE) with k-space weighted image contrast (KWIC) for dynamic gadoxetic acid (Gd-EOB-DTPA)-enhanced MRI of the liver: advantages over Cartesian VIBE in the arterial phase. Eur Radiol.

[11] Kierans A, Parikh N, Chandarana H (2015). Recent advances in MR hardware and software. Radiol Clin North Am.

[12] Chandarana H, Block TK, Rosenkrantz AB, Lim RP, Kim D, Mossa DJ, Babb JS, Kiefer B, Lee VS (2011). Free-breathing radial 3D fat-suppressed T1-weighted gradient echo sequence: a viable alternative for contrast-enhanced liver imaging in patients unable to suspend respiration. Invest Radiol.

[13] McGraw KO, Wong SP (1996). Forming interferences about some intraclass correlation coefficients. Psychol Methods.

[14] Landis JR, Koch GG. The measurement of observer agreement for categorical data. Biometrics. 1977:159–174. 843571

[15] Feng L, Axel L, Chandarana H, Block KT, Sodickson DK, Otazo R (2016). XD-GRASP: golden-angle radial MRI with reconstruction of extra motion-state dimensions using compressed sensing. Magn Reson Med.

[16] Armstrong T, Ly KV, Murthy S, Ghahremani S, Kim GHJ, Calkins KL, Wu HH (2018). Free-breathing quantification of hepatic fat in healthy children and children with nonalcoholic fatty liver disease using a multi-echo 3-D stack-of-radial MRI technique. Pediatr Radiol.

[17] Pruessmann KP, Weiger M, Börnert P, Boesiger P (2001). Advances in sensitivity encoding with arbitrary k-space trajectories. Magn Reson Med.

[18] Victoria T, Jaramillo D, Roberts TPL, Zarnow D, Johnson AM, Delgado J, Rubesova E, Vossough A. Fetal magnetic resonance imaging: jumping from 1.5 to 3 tesla (preliminary experience). Pediatr Radiol. 2014;44:376–386. 10.1007/s00247-013-2857-0 24671739

[19] da Silva Jr NA, Vassallo J, Sarian LO, Cognard C, Sevely A (2018). Magnetic resonance imaging of the fetal brain at 3 Tesla: preliminary experience from a single series. Medicine..

[20] Inaoka T, Sugimori H, Sasaki Y, Takahashi K, Sengoku K, Takada N, Aburano T (2007). VIBE MRI for evaluating the normal and abnormal gastrointestinal tract in fetuses. Am J Roentgenol.

